# Development of Mouse Hepatocyte Lines Permissive for Hepatitis C Virus (HCV)

**DOI:** 10.1371/journal.pone.0021284

**Published:** 2011-06-22

**Authors:** Hussein Hassan Aly, Hiroyuki Oshiumi, Hiroaki Shime, Misako Matsumoto, Taka Wakita, Kunitada Shimotohno, Tsukasa Seya

**Affiliations:** 1 Department of Microbiology and Immunology, Hokkaido University Graduate School of Medicine, Sapporo, Hokkaido, Japan; 2 Department of Virology II, National Institute of Infectious Diseases, Shinjuku, Tokyo, Japan; 3 Research Institute, Chiba Institute of Technology, Narashino, Chiba, Japan; Centre de Recherche Public de la Santé (CRP-Santé), Luxembourg

## Abstract

The lack of a suitable small animal model for the analysis of hepatitis C virus (HCV) infection has hampered elucidation of the HCV life cycle and the development of both protective and therapeutic strategies against HCV infection. Human and mouse harbor a comparable system for antiviral type I interferon (IFN) induction and amplification, which regulates viral infection and replication. Using hepatocytes from knockout (ko) mice, we determined the critical step of the IFN-inducing/amplification pathways regulating HCV replication in mouse. The results infer that interferon-beta promoter stimulator (IPS-1) or interferon A receptor (IFNAR) were a crucial barrier to HCV replication in mouse hepatocytes. Although both IFNARko and IPS-1ko hepatocytes showed a reduced induction of type I interferons in response to viral infection, only IPS-1-/- cells circumvented cell death from HCV cytopathic effect and significantly improved J6JFH1 replication, suggesting IPS-1 to be a key player regulating HCV replication in mouse hepatocytes. We then established mouse hepatocyte lines lacking IPS-1 or IFNAR through immortalization with SV40T antigen. Expression of human (h)CD81 on these hepatocyte lines rendered both lines HCVcc-permissive. We also found that the chimeric J6JFH1 construct, having the structure region from J6 isolate enhanced HCV replication in mouse hepatocytes rather than the full length original JFH1 construct, a new finding that suggests the possible role of the HCV structural region in HCV replication. This is the first report on the entry and replication of HCV infectious particles in mouse hepatocytes. These mouse hepatocyte lines will facilitate establishing a mouse HCV infection model with multifarious applications.

## Introduction

Chronic hepatitis C virus (HCV) infection is a major cause of mortality and morbidity throughout the world infecting around 3.1% of the world's population [1]. The development of much needed specific antiviral therapies and an effective vaccine has been hampered by the lack of a suitable small animal model. The determinants restricting HCV tropism to human and chimpanzee hosts are unknown. Replication of HCV strain JFH1 has been demonstrated in mouse cells only upon antibody selection [2], highlighting the very limited replication efficiency. Human CD81 and occludin have been implicated as important entry receptors for retrovirus particles bearing HCV glycoproteins, HCV pseudoparticles (HCVpp), into NIH3T3 murine cells [3]. However, HCV infection, spontaneous replication and particle production by mouse cells have not yet been reported.

In mammalian cells, the host detects and responds to infection by RNA-viruses, including HCV, by primarily recognizing viral RNA through several distinct pathogen recognition receptors (PRRs), including the cell surface and endosomal RNA sensors Toll-like receptors 3 and 7 (TLR3 and TLR7), and the cytoplasmic RNA sensors retinoic acid-inducible gene I (RIG-I) and melanoma differentiation associated gene 5 (MDA5) [4]. The detection of virus infection by these receptors leads to the induction of interferons (IFNs) and their downstream IFN-inducible anti-viral genes through distinct signaling pathways [5]. Type I IFN is an important regulator of viral infections in the innate immune system [6]. Another type of IFN, IFN-lambda, affects the prognosis of HCV infection, and its response to antiviral therapy [7], [8].

Mutations impairing the function of the RIG-I gene and the induction of IFN were essential in establishing HCV infectivity in human HuH7.5 cells [9]. Similarly, the HCV-NS3/4a protease is known to cleave IPS-1 adaptor molecule, inducing further downstream blocking of the IFN-inducing signaling pathway [10]. These data clearly demonstrate that the host RIG-I pathway is crucial for suppressing HCV proliferation in human hepatocytes. Using a similar strategy, we investigated whether suppressing the antiviral host innate immune system conferred any advantage on HCV proliferation in mouse hepatocytes. We examined the possibility of HCV replication in mice lacking the expression of key factors that modulate the type I IFN-inducing pathways. Only gene silencing of the IFN receptor (IFNAR) or IPS-1 was sufficient to establish spontaneous HCV replication in mouse hepatocytes. To establish a cell line permissive for HCV replication, which is required for further *in vitro* studies of the HCV life cycle in mouse hepatocytes, we immortalized IFNAR- and IPS-1-knockout (ko) mice hepatocytes with SV40 T antigen. Upon expression of the human (h)CD81 gene, these newly established cell lines were able to support HCV infection for the first time in mouse hepatocytes. Viral factors required for HCV replication in mouse hepatocytes were also analyzed.

## Results

### IPS-1-mediated IFN signaling is important for HCV replication in mouse hepatocytes

**Figure 1:**
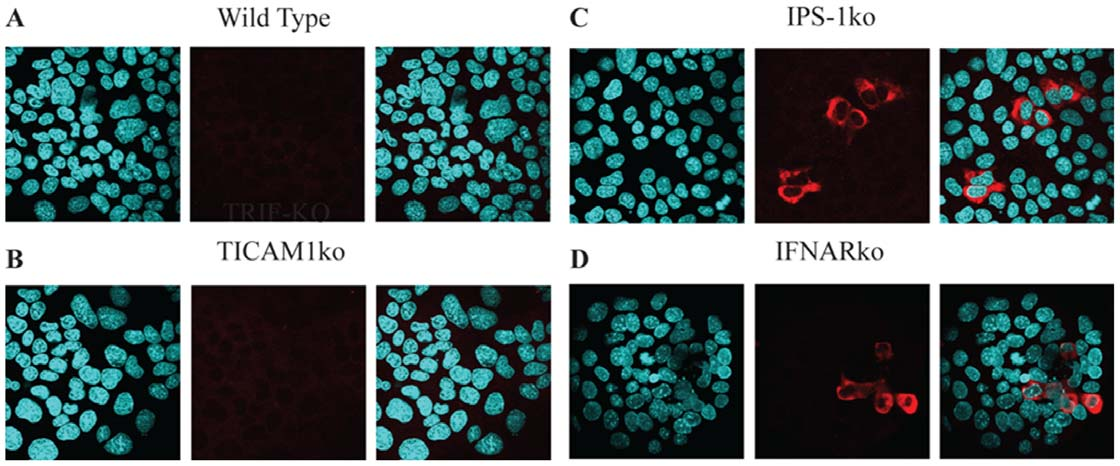
IF detection of of J6JFH1 proteins' expression 5 days after transfection of J6JFH1-RNA through electroporation into wild type (A), TICAM-1ko (B), IPS-1ko (C), and IFNARko (D), freshly isolated primary hepatocytes. A highly sensitive polyclonal antibody extracted from HCV-patient serum (AbS3) was used for the detection. Staining of the uninfected hepatocytes from different Ko mice was also performed and they showed negative for HCV proteins (data not shown).

As a first step in establishing HCV infection in mice, we tested the susceptibility of mouse hepatocytes to persistent expression of HCV proteins after RNA transfection. *In vitro* transcribed chimeric J6JFH1 RNA, in which the HCV structural and non-structural regions were from J6 and JFH1 isolates respectively, was transfected into hepatocytes from wild-type mice. We used a highly sensitive polyclonal antibody derived from HCV-patient serum for the detection of HCV proteins. No HCV proteins were detected five days after transfection (Fig. 1 A), suggesting that wild-type mouse hepatocytes were unable to maintain HCV replication. We then tried to find and block the pathway used by mouse hepatocytes for the detection of viral-RNA and the induction of IFN response. Mouse hepatocytes did not show the expression of either TLR3 or TLR7 as detected by RT-PCR, unlike IPS-1 and RIG-I which was fairly detected (Fig. S1), suggesting that the cytoplasmic RIG-I/IPS-1 pathway is the main pathway utilized by mouse hepatocytes for the detection of RNA viruses. We then checked the susceptibility of hepatocytes from TICAM-1ko, IPS-1ko and IFNARko mice to the prolonged expression of HCV proteins (Fig. 1B–D). Only IPS-1- and IFNARko mouse hepatocytes showed expression of J6JFH1 proteins five days after transfection (Fig. 1), indicating the importance of impaired IPS-1 and/or IFNAR receptors for HCV persistence. Similarly, the detection of the J6JFH1-RNA in transfected hepatocyte lines from various knockout mice showed higher levels in IPS-1 or IFNAR knockout cells compared to TICAM-1knockout cells in which a rapid decline of J6JFH1-RNA levels was noticed similar to the non-replicating control JFH1GND construct (Fig. S2). These data clearly suggest that the RIG-I/IPS-1 but not TLR3/TICAM-1 is the main pathway utilized for the detection of HCV-RNA and the induction of anti-viral immune response in mouse hepatocytes. Its suppression significantly improves HCV replication in mouse hepatocytes.

### Establishment and characterization of immortalized mouse hepatocyte cell lines lacking expression of the IFNAR or IPS-1 gene

**Figure 2:**
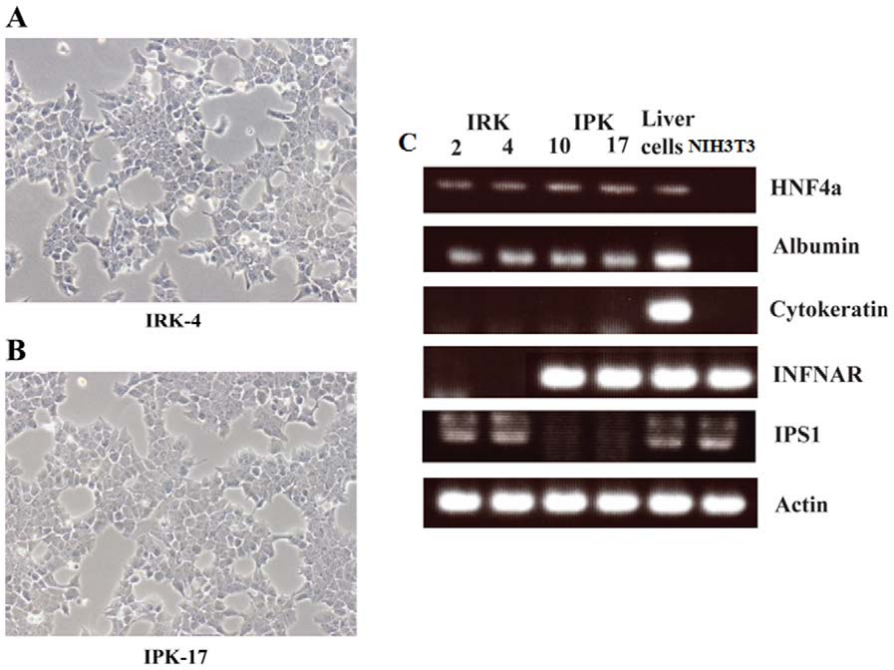
Morphological characteristics of IRK-4 (A) and IPK-17 (B) cells. (C) RT analysis for the expression of albumin, HNF4, cytokeratin, interferon A receptor, and IPS-1 in 2 IFNAR-KO cell lines (IRK2 and 4), 2 IPS-1-KO cell lines (IPK-10 and 17), total liver, and NIH3T3 cells.

We further established mouse hepatocyte lines with disrupted IFNAR or IPS-1 genes through immortalization with SV40T antigen, and used these cell lines to study factors required for the HCV life cycle. Hepatocytes were transduced with SV40T-expressing lentivirus vectors. Six weeks after transduction, hepatocytes transduced with SV40T showed continuous proliferation and clonally proliferating hepatocyte lines were selected. SV40T-immortalized IFNARko and IPS-1ko clones were designated IRK (Fig. 2 A) and IPK (Fig. 2 B), respectively. 20 IRK and 19 IPK clones were picked up, of which IRK clones 2 and 4 (IRK2 and IRK4) and IPK clones 10 and 17 (IPK10 and IPK17) were most closely related to primary mouse hepatocytes in term of differentiation (Fig. 2 C) and were used in the following experiments. Expression of SV40T was confirmed by RT-PCR analysis (data not shown). IRK2, IRK4, IPK10 and IPK17, but not the non-hepatocytic NIH3T3 cells, displayed albumin and hepatocyte nuclear factor 4 (HNF4) expression similar to that observed in liver tissue, but did not express the bile duct marker, cytokeratin. IRK and IPK cells did not show expression of IFNAR and IPS-1 respectively (Fig. 2 C).

### Replication of the HCV genome in IRK and IPK cells

**Figure 3:**
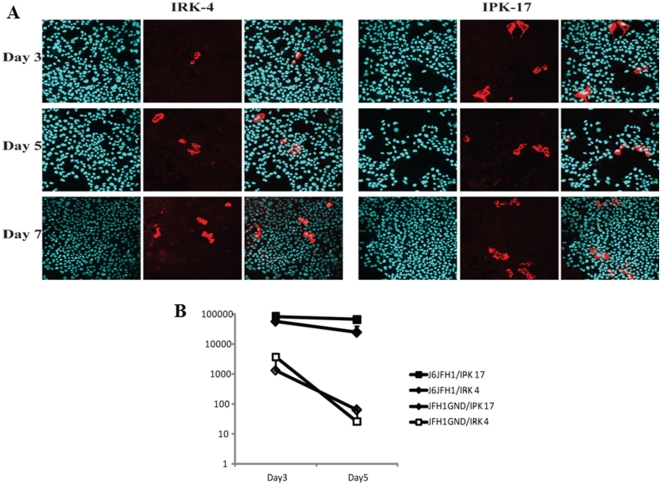
Proliferation of HCV in IRK4 and IPK17 cells over time as detected by immunofluorescence staining of NS5a protein using the CL1 rabbit polyclonal antibody (A) and by quantitative real-time RT-PCR analysis of HCV-RNA levels (B). JFH1GND was used as a negative control to exclude non replicating HCV-RNA. The data plotted represent the average +/− STD of 3 different experiments.

To assess the permissiveness of the established cell lines to HCV replication, we transduced IRK4 and IPK17 cells with J6JFH1 RNA and monitored the HCV protein and RNA levels by IF (Fig. 3 A) and real time RT-PCR (Fig. 3 B). The number of cells expressing HCV proteins, as detected by IF, increased over time, indicating the continuous proliferation of J6JFH1 in these cells. However, the ratio between infected and non-infected cells did not significantly change over time for 7 days after transfection. Similarly, the amount of total J6JFH1 RNA in 1 µg of total cellular RNA was reasonably constant. By contrast, the level of JFH1GND RNA carrying a mutation in NS5B hampering HCV replication, rapidly declined, indicating the requirement of continuous HCV replication for the maintenance of HCV positivity in the transfected mouse hepatocytes. Similar data were obtained from IRK2 and IPK10 cells (data not shown).

### IPS-1-dependent/Interferon-independent pathway is responsible for HCV's cytopathic effect

**Figure 4:**
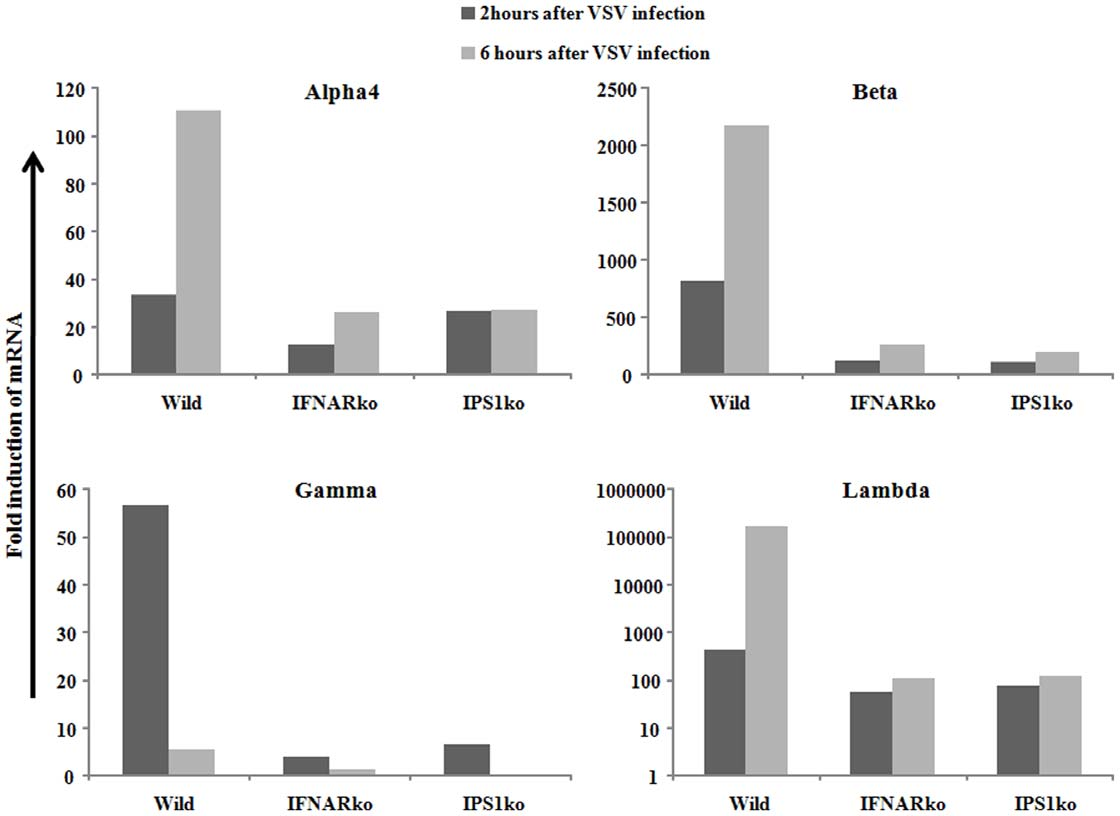
Wild type, IFNARko, and IPS-1ko mice hepatocytes were infected with mock or VSV virus, 2 and 6 hours later, total RNA was extracted from the cells, and interferon alpha, beta, gamma and lambda mRNA induction levels were measured by real-time RT-PCR. Similar results were obtained from 2 different experiments, each was performed in duplicates. The data plotted represent the mean duplicate readings in one of them.

**Figure 5:**
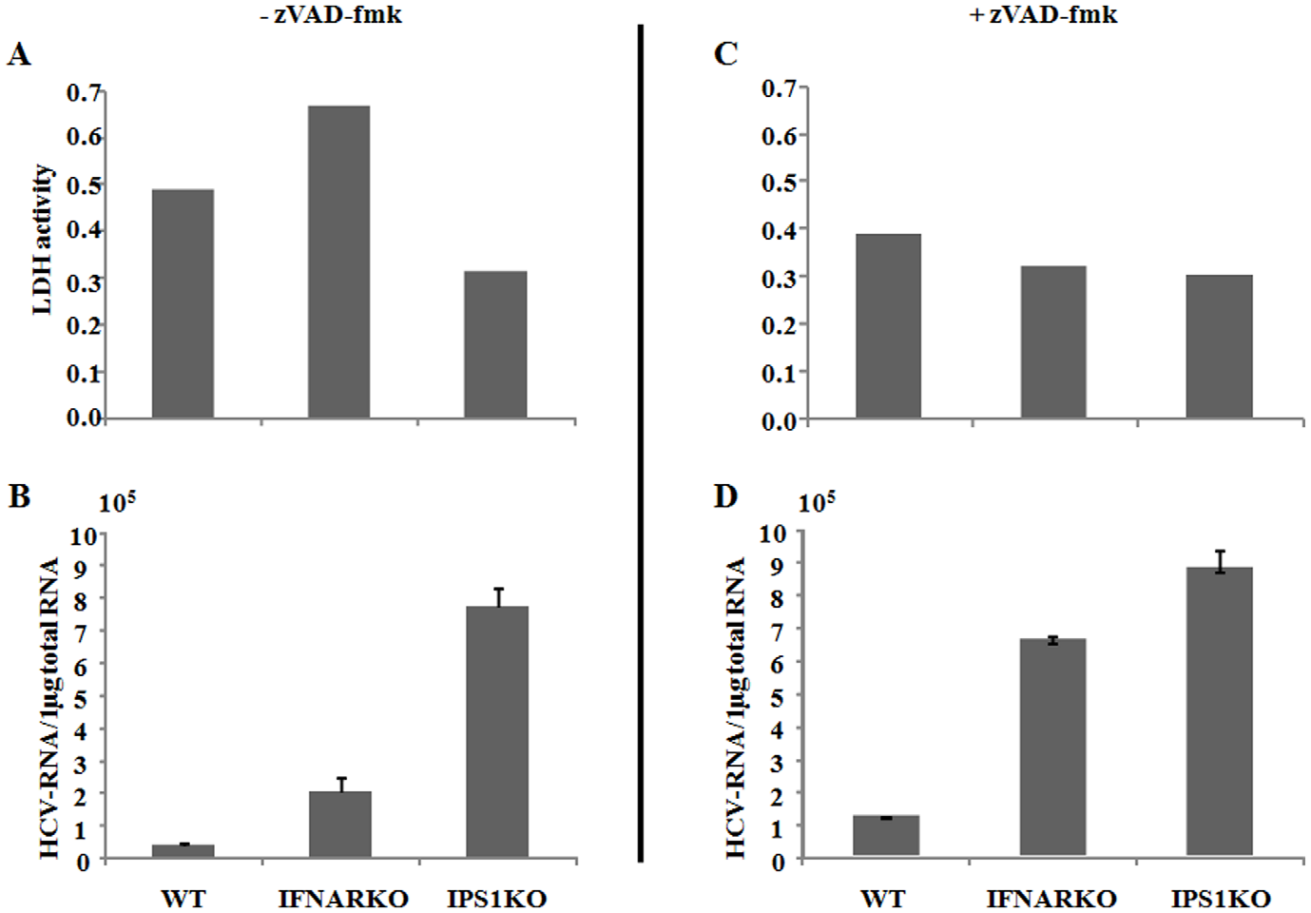
Measurement of J6JFH1 mediated cytopathic effect in wild type, IFNARko, and IPS-1ko mouse hepatocytes. Cultrure medium were left untreated (A;B) or treated with 20 µM of zVAD-fmK (C;D) 2 days before and after J6JFH1-RNA transfection. One day after transfection of J6JFH1-RNA, culture medium was discarded and cells were washed with PBS. A new medium was added and cells were cultured for another 24 hours. The LDH activity in the culture medium was measured in 2 different experiments in duplicates and showed similar results, the average levels of a duplicate from a single experiment was plotted (A, C). HCV-RNA titers in the cells were also measured using real-time RT-PCR (B, D), the data shown represent the mean +/− STD of 3 different experiments.

In comparison to IPS-1ko hepatocytes, J6JFH1-RNA in IFNARko were lower and decreased further after its transfection, while higher stable levels of J6JFH1-RNA were maintained in IPS-1ko cells (Fig. 3 B and Fig. S2). Similarly, larger numbers of HCV-positive cells were detected in IPS-1ko hepatocytes compared with their IFNARko counterparts (Fig. 3 A), suggesting that the IPS-1 disruption benefits HCV replication in a distinct manner from IFNAR disruption. To measure the interferon induction after RNA virus infection in those cells, we used a highly infectious RNA-Virus (VSV) and measured the induction of interferon after its infection. All the interferons measured showed similar suppression of induction in IFNARko and IPS-1ko hepatocytes (Fig. 4). Surprisingly, cellular cytopathic effect that was monitored after transfection of J6JFH1-RNA was markedly reduced in IPS-1ko but not in IFNARko hepatocytes after transfection (Fig. 5A). This suppression was accompanied by an increase of J6JFH1-RNA levels in IPS-1ko cells, suggesting that minimal cellular damage induced by HCV replication in IPS-1-/- cells led to the improvement of HCV proliferation in mouse hepatocytes (Fig. 5B).Reduction of HCV-induced cellular cytotoxicity (Fig.5C), and improvement of HCV replication (Fig.5D) in wild type, and IFNAR-KO cells were found when we cultured the cells with a pan-caspase inhibitor, zVAD-fmk, 2 days before and after HCV-RNA transfection. We reasoned that the IPS-1 pathway rather than the IFNAR pathway capacitates hepatocytes to induce HCV-derived apoptotic cell death and its disruption resulted in the circumvention of cell death.

### Human CD81 is required for HCV infection of mouse hepatocytes

**Figure 6:**
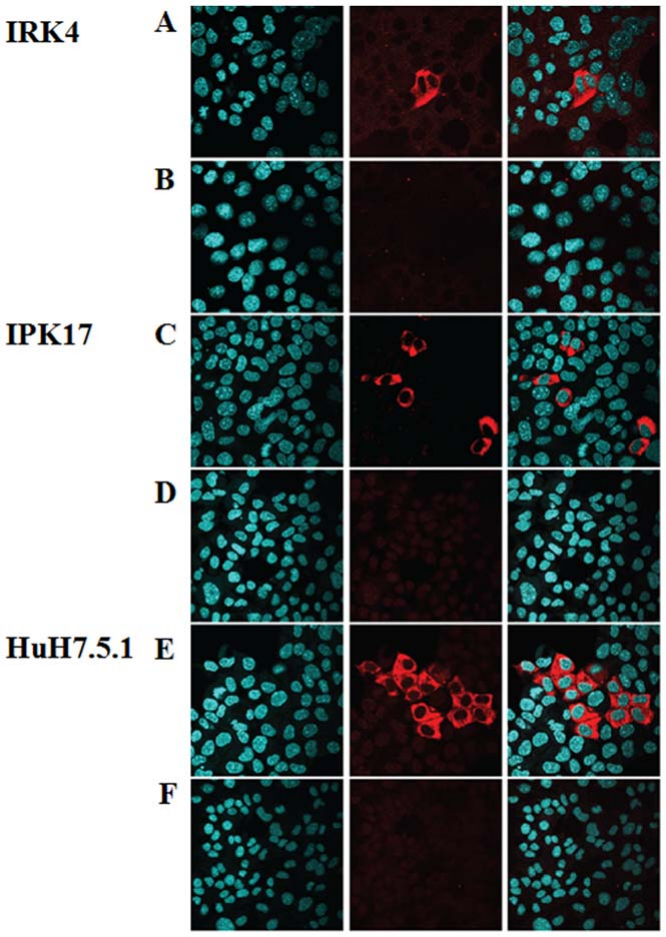
J6JFH1 infection into IRK-4 and IPK17 cells. HCV-NS5A protein detection in mouse IRK4 (A,B) and IPK17 (C,D) and human 7.5.1 cells (E,F). The cells were transduced with lentivirus expressing human CD81 gene at 10 MOI. 48 hours later the cells were infected with 100 times concentrated supernatant medium, collected during 1 week after transfection of HuH7.5.1 cells with J6JFH1-RNA (A, C, and E) or JFH1GND-RNA (B, D, and F).

Similar to the primary mouse hepatocytes, immortalized mouse hepatocytes showed the expression of all the mouse counterparts of human HCV entry receptors (Fig. S3). Human CD81 and hOccludin, but not other human HCV receptors such as SR-B1 or claudin1, have previously been reported to be essential for HCVpp entry into NIH3T3 mouse cells [3]. We then expressed hCD81 and/or hOccludin in IRK2 and IRK4 cells using lentivirus vectors. Using a MOI of 10, 95% transfection efficiency was achieved (Fig. S4) with lentivirus vector. We next tested the effect of these proteins on HCV particle (HCVcc) infection. Human CD81 alone was found to be required for J6JFH1 infection into all IRK and IPK cells tested (Fig. S5 and Fig. 6 A, and B). For the first time in mouse hepatocytes, HCV proteins were detected in nearly 1% of the cells used for infection. These data demonstrated the importance of hCD81 in establishing HCVcc infection in mouse hepatocytes.

### Viral factors affecting HCV replication in mouse hepatocytes

**Figure 7:**
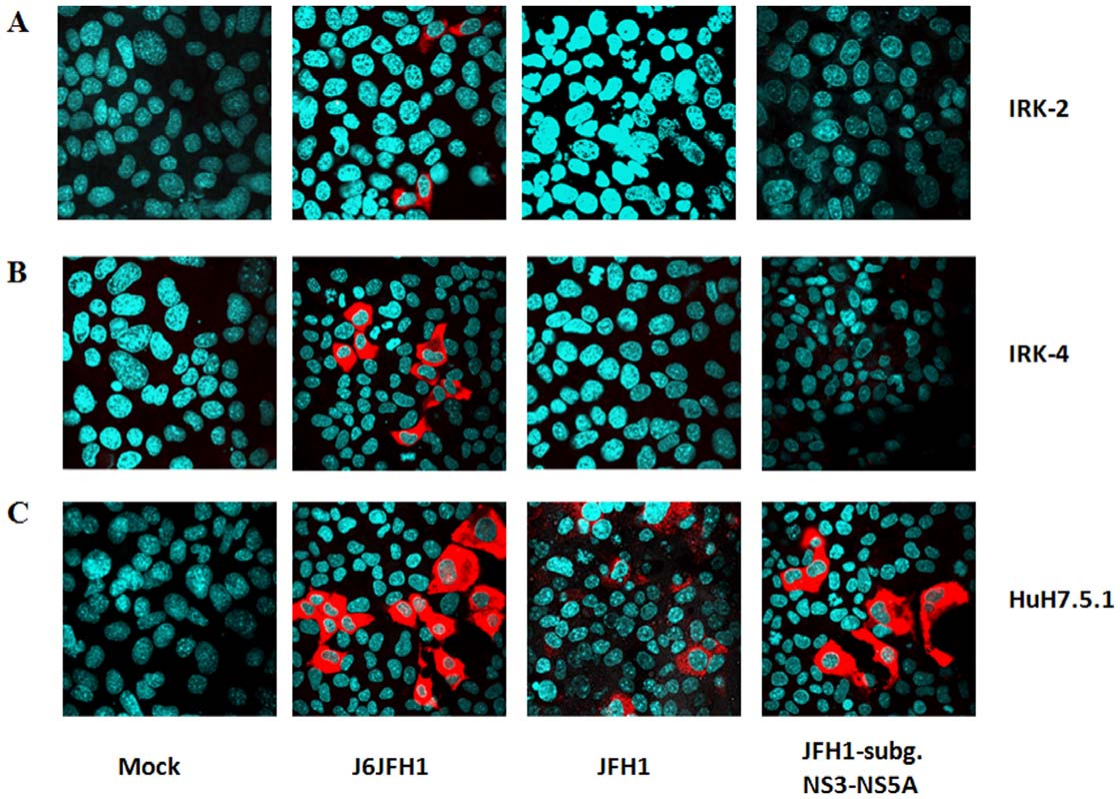
Detection of HCV-NS5A protein in IRK-2 (A), IRK-4 (B) and HuH-7.5 cells (C) by IF 5 days after transfection with J6JFH1, FL-JFH1 or subgenomic JFH1-RNA.

After successfully establishing J6JFH1 infection in mouse hepatocytes, we attempted to infect these cells with other strains of HCV. Human CD81-expressing IPK17 cells were infected with full-length JFH1FL, however, no infection was detected (data not shown). This might be due to a problem in infection and/or replication. We further examined the replication efficiency of JFH1FL, the subgenomic JFH1 replicon and the J6JFH1 chimera in two different mouse hepatocyte lines and the HuH7.5.1 cell line. The persistent expression of HCV proteins was detected seven days after RNA transfection. Although HCV proteins were detected in HuH7.5.1 cells in all cases (Fig. 7 C), only J6JFH1 proteins were detected in the mouse hepatocyte lines, suggesting for the first time the importance of the J6 structural region for the replication of HCV in mouse hepatocytes (Fig. 7 A, and B).

## Discussion

Gene silencing of either IPS-1 or IFNAR significantly improves HCV replication and persistence in mouse hepatocytes compared with wild-type or TICAM-1ko mice. This result demonstrated the importance of the IPS-1 pathway rather than the TICAM-1 pathway in the induction of type I IFN by HCV infection, and revealed that the IFNAR amplification pathway confers resistance to HCV in mouse hepatocytes independently of TICAM-1. In accordance with our data, HCV-NS3/4A protease is known to cleave the IPS-1 and/or RIG-I-complement molecules including DDX3 and Riplet in humans to overcome the host innate immune response, showing the importance of RIG-I/IPS-1 pathway suppression in the establishment of HCV infection [10], [11], [12].

To further study factors affecting the HCV life cycle in mouse hepatocytes, we established IPK and IRK immortalized mouse hepatocyte lines by transduction with SV40T antigen. The established hepatocytes cell lines showed expression of HNF4, a major hepatocyte transcription factor, required for hepatocyte differentiation and liver-specific gene expression [13]. The maintenance of hepatocellular functions was demonstrated by continuous expression of hepatocyte specific differentiation marker, albumin, and the lack of expression of the bile duct marker, cytokeratin. The close resemblance of these cell lines to primary mouse hepatocytes is crucial to ensure the physiological relevance of factors identified in these cell lines that affect the HCV life cycle.

It is worth noting that HCV replication in IPS-1ko was higher than that in IFNARko hepatocytes. Since IPS-1 is present upstream of IFNAR in the IFN-amplification pathway, this higher J6JFH1 replication efficiency in IPS-1ko hepatocytes suggested the presence of an additive factor affecting HCV replication other than the induction of IFNAR-mediated type I IFN. This enhanced replication efficiency was also not accompanied by the induction of other interferon types, but was correlated with the reduction of HCV-induced apoptosis in mouse hepatocytes. This data clearly demonstrates that IPS-1 is playing an important role in the regulation of HCV infection in mouse hepatocytes through two different pathways, the IFN-induction pathways and another new IFN-independent pathway, leading to apoptotic cell death and elimination of HCV-harboring hepatocytes. The cytopathic effect of HCV infection in human cells is still contradictory. Although, some reports showed the induction of apoptosis and cell death by HCV infection in human hepatocytes [14], [15], [16], others showed suppression of apoptosis by HCV proteins [17], [18]. This difference may be due to the different cell lines used in the different studies. Almost all the studies reporting HCV-induced apoptosis used hepatocellular carcinoma cell lines. Since it has been established that the inability to undergo apoptosis is essential for the development of cancer [19], [20], [21], our use of immortalized, non-cancerous hepatocytes may make it possible to reproduce the physiological response of the cells to HCV infection more closely. The IPS-1 regulation of cell death following the introduction of HCV-RNA may also regulate the effector cell function. It is likely that hepatocyte debris generated secondary to intrinsic production of viral dsRNA in HCV-infected hepatocytes affect the antiviral effector response of the immune system through maturation of dendritic cells [22]. Hence, the effector cell activation may be enhanced by the induction of cell death through the IPS-1 pathway in hepatocytes which may facilitate producing dsRNA-containing debris.

In comparison to the JFH1GND construct with deficient replication that showed a rapid reduction in its RNA levels over time after transfection into mouse hepatocytes, J6JFH1 RNA was detected at four-log higher levels and was maintained at a relatively stable levels in IPS-1ko hepatocytes. Although the number of mouse cells expressing HCV proteins was found to increase over time, as detected by IF, the ratio between HCV-negative and -positive cells did not show any significant change for 7 days after transfection and increased after 10 days (data not shown). This indicates a negative selection of HCV-bearing cells over time which may be due to slower cellular replication, or loss of HCV replication. Another possibility may be that HCV infection is affected by the presence of an inhibitory factor possibly triggered by HCV replication or the lack of a human host factor required for HCV replication. Due to the initial replication of HCV in the transfected IPK and IRK mouse hepatocytes for the first 7 days and the establishment of infection, we favor the presence of a possible inhibitory factor that may be triggered by HCV replication. Another factor that also limits HCV spread in mouse hepatocytes is the failure of HCV to produce infectious particles in these cells (data no shown).

Using this newly established immortalized mouse hepatocyte line, we found that although J6JFH1, JFH1FL and the subgenomic JFH1 replicon all share a similar non-structural region derived from isolate JFH1 that is required for HCV replication, and although all of these constructs can replicate efficiently in HuH7.5.1 cells, strikingly, only J6JFH1 carrying the J6 structural region replicated in mouse hepatocytes. This indicates the importance of the J6 structural region and/or the chimeric construct between J6 and JFH1 for HCV replication in mouse hepatocytes. Structural regions are known to be important for HCV entry and/or particle formation [23], but this is the first time that their importance in replication in HCV-bearing cells has been demonstrated. This finding clearly shows the importance of non-hepatoma cell lines with less genetic abnormalities and mutations for the discovery of new aspects of the life cycle of HCV.

Although, the co-expression of human CD81 and Occludin genes was found to be important for HCVpp entry into murine NIH3T3 cells [3], the expression of hCD81 alone was sufficient for J6JFH1 entry into mouse hepatocytes. This may be explained by the different cell lines used in the different studies. In contrast to NIH3T3 cells, we used immortalized hepatocytes that showed close physiological resemblance to primary mouse hepatocytes and showed the expression of all the mouse counterparts of HCV entry receptors. A study from a different group showed that adaptive mutations in HCV envelope proteins allowing its interaction with murine CD81 is enough for efficient HCVpp entry without the expression of any human entry receptors in murine cells [24]. This report, together with ours, suggest that CD81 is the main human host restriction factor for HCV entry, and that overcoming this problem either by HCV adaptation to murine CD81, or the expression of human CD81 in murine hepatocytes is essential for HCV entry. Although our lentivirus transfection efficiency with CD81 was around 95% in IPK and IRK clones, only 1% of the cells were prone to infection with HCVcc. Also, HCVpp showed lower entry levels in those cells compared to HuH7.5.1 cells (Fig. S6). This suggests that hCD81 expression is the minimum and most crucial requirement for HCV entry into mouse hepatocytes. The discovery and expression of other co-receptors facilitating HCV entry in human cells is still required for efficient and robust HCV infection.

In summary, the suppression of IPS-1 is important for the establishment of HCV infection and replication in mouse hepatocytes through the suppression of both interferon induction and interferon independent J6JFH1-induced cytopathic effect. We have established hepatocytes lines from IPS-1 and IFNARko mice that support HCV replication and infection. These cell lines will be very useful in identifying other species restriction factors and viral determinants required for further establishment of a robust and efficient HCV life cycle in mouse hepatocytes. Using those cells, we showed for the first time the importance of HCV structural region for viral replication. IRF3ko mouse embryo fibroblasts (MEFs) were previously shown to support HCV replication more efficiently than wild MEFs [25]. Since the knockout of IPS-1 mainly suppresses signaling in response to virus RNA detection, and maintains an intact IFN response to other stimulants, it may result in minimum interference to adaptive immune responses as compared to IRF3 or IFNARko. Therefore, further development of hCD81-transgenic IPS-1ko mice may serve as a good model for the study of immunological responses against HCV infection. This mouse model can be used as a backbone for any further future models supporting robust HCV infectivity for the study of HCV pathogenesis, propagation and vaccine development.

## Material and Methods

### Cell culture

HuH7.5.1 cells were cultured in high-glucose Dulbecco's modified Eagle's medium (DMEM; Gibco/Invitrogen, Tokyo, Japan) supplemented with 2 mM L-glutamine, 100 U of penicillin/ml, 100 µg of streptomycin/ml and 10% fetal bovine serum. Mouse primary hepatocytes were isolated from the liver using collagenase perfusion through the inferior vena cava (IVC), while clamping the animal's intrathoracic extension. Hepatocyte isolation and perfusion control were performed as previously described [26]. Primary and immortalized hepatocytes were cultured in a similar medium supplemented with: HEPES (Gibco/Invitrogen), 20 mmol/L; L-proline, 30 µg/mL; insulin (Sigma, St. Louis, MO, USA), 0.5 µg/mL; dexamethasone (Wako, Osaka, Japan), 1×10^−7^ mol/L; NaHCO_3_, 44 mmol/L; nicotinamide (Wako), 10 mmol/L; EGF (Wako), 10 ng/mL; L-ascorbic acid 2-phosphate (Wako), 0.2 mmol/L; and MEM-non essential amino acids (Gibco/Invitrogen), 1%.

### Gene-disrupted mice

All mice were backcrossed with C57BL/6 mice more than seven times before use. Toll-like receptor adaptor molecule 1 (TICAM-1) ko [27] and IPS-1ko mice [28] were generated in our laboratory (detailed information regarding the IPS-1 mice will be presented elsewhere). All mice were maintained under specific-pathogen-free conditions in the animal facility of the Hokkaido University Graduate School of Medicine (Japan).

### RNA extraction, reverse transcriptase polymerase chain reaction (RT-PCR) and real-time RT-PCR

RNA was extracted from cultured cells using Trizol reagent (Invitrogen, San Diego, CA, USA) according to the manufacturer's protocol. Using 1 µg of total RNA as a template, we performed RT-PCR and real-time RT-PCR as previously described [29], [30].

### *In vitro* RNA transcription, transfection and preparation of J6JFH1 and Jfh1 viruses

*In vitro* RNA transcription, transfection into HuH7.5.1 or mouse hepatocytes, and preparation of J6JFH1 and JFH1 viruses, were all performed as previously reported [31]. RNA transfection into human and mouse hepatocytes was performed by electroporation using a Gene Pulser II (Bio-Rad, Berkeley, California) at 260 V and 950 Cap.

### HCV infection

J6JFH1 and JFH1 concentrated medium were adjusted to contain a similar RNA copy number by real-time RT-PCR. 2×10^4^ cells/well were cultured in 8-well glass chamber slides. After 24 hours, the medium was removed and replaced by concentrated medium containing JFH1 or J6JFH1 viruses. After three hours, the concentrated medium was removed, cells were washed with PBS and incubated in fresh medium for 48 hours, before the detection of infection.

### Lentivirus construction, titration and infection

The gene encoding T antigen from simian virus was cloned from plasmid CSII-EF-SVT [32]. The genes encoding human CD81 and occludin were cloned from HuH-7.5.1 cells using the Zero Blunt TOPO PCR Cloning Kit (Invitrogen) according to the manufacturer's protocol. These genes were then inserted into the GFP reporter gene-containing lentiviral expression (pLBIG) vector using the *Eco*RI and *Xho*I restriction sites for SV40T and hCD81, and the *Xba*I and *Xho*I restriction sites for hOccludin. Lentivirus expression vectors were then constructed as previously described [27]. GFP expression was used for the titration of lentivirus vectors, and a multiplicity of infection (MOI) of 10 was used for the infection of mouse cells. Forty-eight hours after the transfection of hCD81 and/or hOccludin, cells were trypsinized and counted. Then, 2×10^4^ cells/well were cultured in 8-well glass chamber slides for HCV infection and 5×10^4^ cells/well were cultured in 12-well plates, along with 1 ml of medium containing HCVpp, for HCV entry experiments.

### HCVpp construction and the detection of luciferase expression

HCVpp containing the E1 and E2 proteins from HCV isolate J6 and expressing the luciferase reporter gene were a kind gift from Dr. Thomas Pietschmann at the TWINCORE Center for Experimental and Clinical Infection Research, Germany. The production of HCVpp and the measurement of luciferase levels were performed as previously described [33].

### Indirect immunofluorescence (IF)

IF expression of HCV proteins was detected in the infected cells using antibodies in the serum of chronic HCV patients or rabbit IgG anti-NS5A antibody (Cl-1) (both kind gifts from K. Shimotohno, Chiba Institute of Technology, Japan). Goat anti-human IgG Alexa 594 and goat anti-rabbit Alexa 594 (Invitrogen) were used as secondary antibodies, respectively. Fluorescence detection was performed on a ZEISS LSM 510 Meta confocal microscope (Zeiss, Jena, Germany).

### Detection of cell death

Culture medium was collected from HCV infected and control cells and used for measuring lactate dehydrogenase (LDH) levels using an LDH cytotoxicity detection kit (Takara Biomedicals, Tokyo, Japan). Light absorbance was then measured according to the manufacturer's protocol.

### Ethic Statement

This study was carried out in strict accordance with the recommendations in the Guide for the Care and Use of Laboratory Animals of the National Institutes of Health. The protocol was approved by the Committee on the Ethics of Animal Experiments in the Animal Safety Center, Hokkaido University, Japan. All mice were used according to the guidelines of the institutional animal care and use committee of Hokkaido University, who approved this study as ID number: 08-0243, “ Analysis of immune modulation by toll-like receptors”.

## Supporting Information

Figure S1 RT detection of TLR3, TLR7, RIG-I, and IPS-1 expression in mouse hepatocytes. GAPDH expression was used as internal control, and RNA from CD11c+ spleenocytes (dendritic cells) was used as positive control.
(TIF)

Figure S2 Proliferation of HCV in IPS-1, TICAM-1(TRIF) and IFNAR-knockout mouse hepatocytes over time as detected by quantitative real-time RT-PCR analysis of HCV-RNA levels. JFH1GND transfection into IPS-1 knockout cells was used as a negative control to exclude non replicating HCV RNA. The data plotted represent the average +/− STD of 3 different experiments.
(TIF)

Figure S3 RT detection of CD81, Occludin, Claudin 1, SRB1, and LDL receptor expression in primary, IRK4 and IPK17 mouse hepatocytes. GAPDH expression was used as internal control.
(TIF)

Figure S4 Estimation of the transfection efficiency of lentivirus vector expressing green fluorescent protein (GFP) as a reporter, together with hCD81 or hOccludin. 48 hours after transfection with the lentivirus vector, cells were trypsinized and GFP positive cells were detected by BD FACSCalibur (BD Biosciences).
(TIF)

Figure S5 HCV infection of IRK2 cells transfected with lentivirus expressing hCD81 and/or hOccludin. IRK2 cells were transfected with lentivirus expressing empty vector (A), hCD81 (B), hOccludin (C) or hCD81 and hOccludin (D) at a MOI of 10. After 48 hours, the cells were infected with concentrated J6JFH1 transfected 7.5.1 culture medium. After a further three hours, cells were washed with PBS and incubated in fresh medium. After another 48 hours, HCV infection was examined through the detection of HCV-NS5a protein expression by immunofluorescence staining.
(TIF)

Figure S6 HCVpp entry into mouse cells. A similar number of IPK17 and HuH7.5.1 were cultured in triplicate. IPK17 cells were only transfected with lentivirus expressing hCD81, while HuH7.5.1 cells were transfected with empty vector at a MOI of 10. After 48 hours, the medium was replaced with a new medium containing mock VSVG-pp or HCVpp expressing luciferase. After another 48 hours, pseudoparticles entry was determined by measuring the luciferase activity. In order to compare the HCVpp entry between IPK17 and HuH7.5.1 cells, the luciferase expression from VSV-Gpp entry was used an internal control, while that from HCVpp was plotted relatively.
(TIF)
