# Supplementary figures and images for: Development of Mouse Hepatocyte Lines Permissive for Hepatitis C Virus (HCV)
Source: PLoS One. 2011 Jun 22;6(6):e21284. doi: 10.1371/journal.pone.0021284 (PMC3120852; doi:10.1371/journal.pone.0021284)

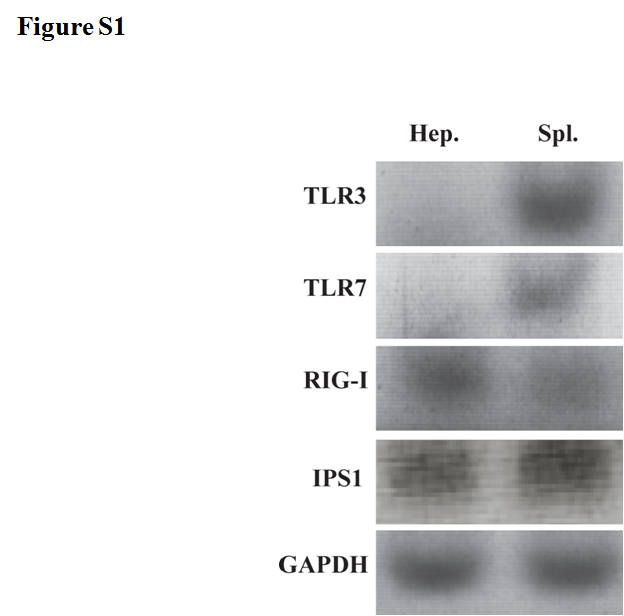

Supplement: Figure S1 — RT detection of TLR3, TLR7, RIG-I, and IPS-1 expression in mouse hepatocytes. GAPDH expression was used as internal control, and RNA from CD11c+ spleenocytes (dendritic cells) was used as positive control. (TIF) [file pone.0021284.s001.tif]

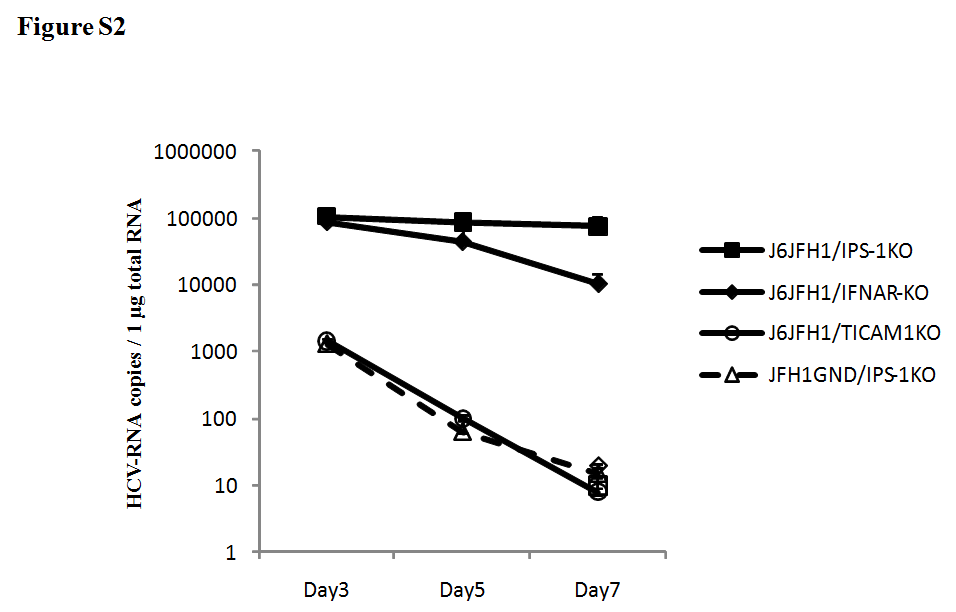

Supplement: Figure S2 — Proliferation of HCV in IPS-1, TICAM-1(TRIF) and IFNAR-knockout mouse hepatocytes over time as detected by quantitative real-time RT-PCR analysis of HCV-RNA levels. JFH1GND transfection into IPS-1 knockout cells was used as a negative control to exclude non replicating HCV RNA. The data plotted represent the average +/− STD of 3 different experiments. (TIF) [file pone.0021284.s002.tif]

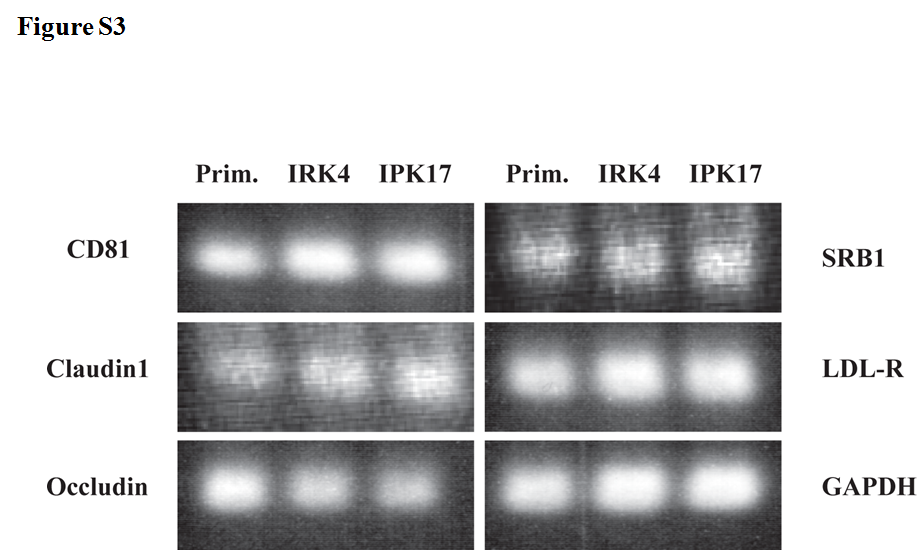

Supplement: Figure S3 — RT detection of CD81, Occludin, Claudin 1, SRB1, and LDL receptor expression in primary, IRK4 and IPK17 mouse hepatocytes. GAPDH expression was used as internal control. (TIF) [file pone.0021284.s003.tif]

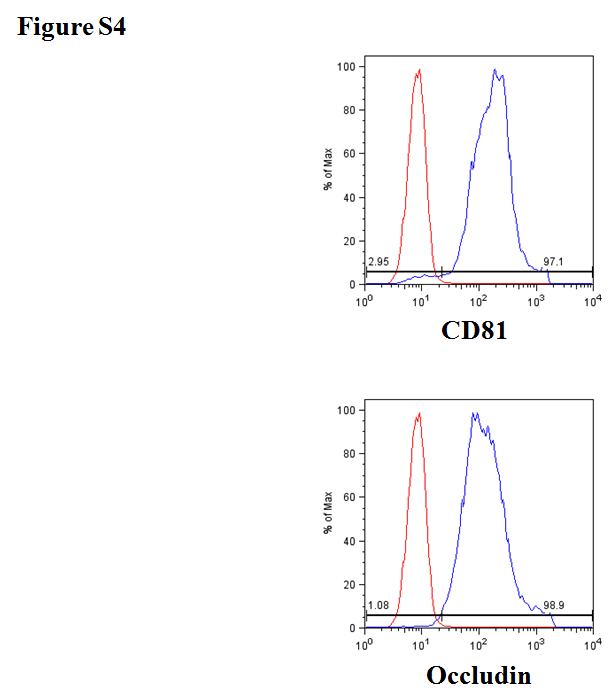

Supplement: Figure S4 — Estimation of the transfection efficiency of lentivirus vector expressing green fluorescent protein (GFP) as a reporter, together with hCD81 or hOccludin. 48 hours after transfection with the lentivirus vector, cells were trypsinized and GFP positive cells were detected by BD FACSCalibur (BD Biosciences). (TIF) [file pone.0021284.s004.tif]

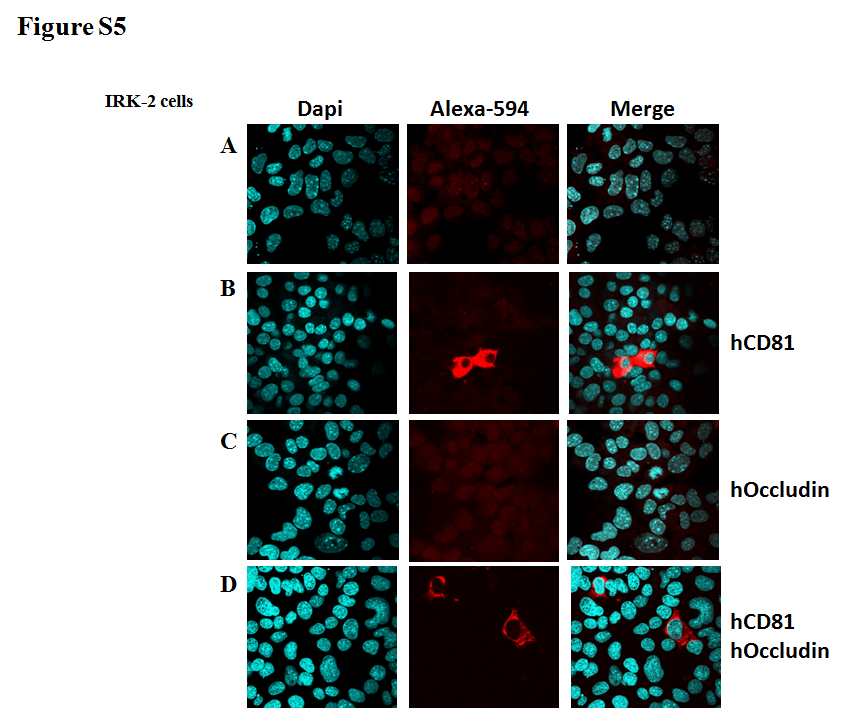

Supplement: Figure S5 — HCV infection of IRK2 cells transfected with lentivirus expressing hCD81 and/or hOccludin. IRK2 cells were transfected with lentivirus expressing empty vector (A), hCD81 (B), hOccludin (C) or hCD81 and hOccludin (D) at a MOI of 10. After 48 hours, the cells were infected with concentrated J6JFH1 transfected 7.5.1 culture medium. After a further three hours, cells were washed with PBS and incubated in fresh medium. After another 48 hours, HCV infection was examined through the detection of HCV-NS5a protein expression by immunofluorescence staining. (TIF) [file pone.0021284.s005.tif]

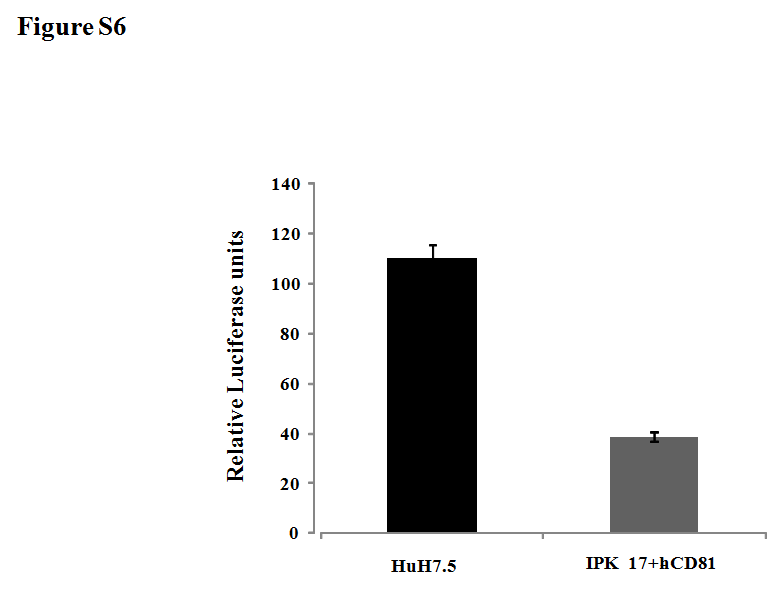

Supplement: Figure S6 — HCVpp entry into mouse cells. A similar number of IPK17 and HuH7.5.1 were cultured in triplicate. IPK17 cells were only transfected with lentivirus expressing hCD81, while HuH7.5.1 cells were transfected with empty vector at a MOI of 10. After 48 hours, the medium was replaced with a new medium containing mock VSVG-pp or HCVpp expressing luciferase. After another 48 hours, pseudoparticles entry was determined by measuring the luciferase activity. In order to compare the HCVpp entry between IPK17 and HuH7.5.1 cells, the luciferase expression from VSV-Gpp entry was used an internal control, while that from HCVpp was plotted relatively. (TIF) [file pone.0021284.s006.tif]
